# Success of Hand Movement Imagination Depends on Personality Traits, Brain Asymmetry, and Degree of Handedness

**DOI:** 10.3390/brainsci11070853

**Published:** 2021-06-25

**Authors:** Elena V. Bobrova, Varvara V. Reshetnikova, Elena A. Vershinina, Alexander A. Grishin, Pavel D. Bobrov, Alexander A. Frolov, Yury P. Gerasimenko

**Affiliations:** 1 Pavlov Institute of Physiology of the Russian Academy of Sciences, 199034 Saint-Petersburg, Russia; 3069@bk.ru (V.V.R.); ver_elen@mail.ru (E.A.V.); grishin-ckb@yandex.ru (A.A.G.); gerasimenko@infran.ru (Y.P.G.); 2 Institute of Translational Medicine of Pirogov of Russian National Research Medical University, 117997 Moscow, Russia; p-bobrov@yandex.ru (P.D.B.); admin@ihna.ru (A.A.F.); 3 Institute of Higher Nervous Activity and Neurophysiology of the Russian Academy of Sciences, 117485 Moscow, Russia; 4 Department of Physiology and Biophysics, University of Louisville, Louisville, KY 40292, USA; 5 Kentucky Spinal Cord Injury Research Center, Frazier Rehab Institute, University of Louisville, UofL Health, Louisville, KY 40202, USA

**Keywords:** motor imagery, brain-computer interface, personality traits, psychology, interhemispheric asymmetry, handedness, neurorehabilitation

## Abstract

Brain-computer interfaces (BCIs), based on motor imagery, are increasingly used in neurorehabilitation. However, some people cannot control BCI, predictors of this are the features of brain activity and personality traits. It is not known whether the success of BCI control is related to interhemispheric asymmetry. The study was conducted on 44 BCI-naive subjects and included one BCI session, EEG-analysis, 16PF Cattell Questionnaire, estimation of latent left-handedness, and of subjective complexity of real and imagery movements. The success of brain states recognition during imagination of left hand (LH) movement compared to the rest is higher in reserved, practical, skeptical, and not very sociable individuals. Extraversion, liveliness, and dominance are significant for the imagination of right hand (RH) movements in “pure” right-handers, and sensitivity in latent left-handers. Subjective complexity of real LH and of imagery RH movements correlates with the success of brain states recognition in the imagination of movement of LH compared to RH and depends on the level of handedness. Thus, the level of handedness is the factor influencing the success of BCI control. The data are supposed to be connected with hemispheric differences in motor control, lateralization of dopamine, and may be important for rehabilitation of patients after a stroke.

## 1. Introduction

The technology of motor imagery-based on BCI has been used as a treatment for rehabilitation of post stroke and spinal cord injury patients as well as of the individuals with musculoskeletal disorders [1,2,3,4,5,6,7,8,9,10,11,12,13,14,15,16,17]. Particular attention is paid to training methods [7,12,18], the use of exoskeletons [9,10,11] and augmented and virtual reality [19,20]. However, the use of BCI in rehabilitation is restricted due to BCI-illiteracy phenomenon: about 10–30% of users cannot control BCI [21]. Finding the predictors of successful BCI control is an important challenge.

The predictors could be EEG characteristics such as: higher amplitude of sensory-motor rhythm in the rest state [22,23,24], theta rhythm in frontal and posterior parietal cortex, gamma rhythm in prefrontal or frontal-parietal cortex, alpha rhythm in all cortical areas [25,26,27].

Personality traits are also shown to be predictors of ability to control BCI (see review [28]). These include memory size and ability to control attention [29]; the ability to concentrate attention, assessed by the attitudes towards work (AHA) test [30,31,32]; degree of self-reliance (Q2) assessed by the 16PF questionnaire [21]; confidence in ability to control BCI and lack of fear of failure [33,34,35,36,37], dealing with technology [34]; activity (active learners tend to perform better than reflexive ones) [21,38]; spatial abilities assessed by the success of mental rotation of figures [21,28,39]; ability to perform precise hand movements assessed by two-hand coordination test [29]; good kinesthetic and visual-motor imagination [40]; ability to imagine (abstractedness, M) in opposite of practicality and down-to-earthness [21]. Additionally, factors such as mood [41], motivation [32,33,41], as well as age (over 25 is better than younger) [42] and gender (women perform better than men) [42] are shown to be predictors of successful BCI control.

Personality traits are known to be connected with EEG-parameters. Neuroticism, estimated by Eysenck’s personality inventory (EPI), correlates with EEG-activity [43,44,45]. According to [43], neuroticism at rest is related to high level of alpha rhythm in the right posterior regions of the brain, and increased frontal asymmetry variability at the mid-frontal sites [44]. Calculation of correlation of EEG-parameters and personality traits, estimated by the Big Five [46,47], demonstrate negative correlation of agreeableness and beta band [48], and openness to experience, agreeableness and conscientiousness correlate with alpha, beta1, beta2, delta, and theta rhythms [49].

There is evidence of interhemispheric differences in correlation of EEG parameters with anxiety [50,51] and with neuroticism [44]. For example, when analyzing the relationship between spectral characteristics of EEG and anxiety (assessed by state-trait anxiety inventory (STAI) and 16PF questionnaire), interhemispheric differences were revealed [51]. In the right but not in the left hemisphere, there were significant positive correlations between anxiety and power of beta2 rhythm (at rest with open eyes). In the left hemisphere, anxiety was negatively correlated with power of alpha rhythm [51].

Thus, there is a lot of information that personality traits correlate with the EEG activity of different parts of the brain in different frequency ranges, and a number of studies indicate the lateralization of brain regions whose activity correlates with personality traits. At the same time, there is no information in BCI studies about how the success of brain state recognition in persons with different traits is connected with interhemispheric asymmetry.

In previous study we demonstrated that the correlation of success of the recognition of brain states during kinesthetic imagination of hand movements in naive right-handed users of BCI with different personality traits depends on whether the right or the left-hand movements are imagined [52].

Different personality traits, which are important for qualitative imagination of the right or the left hand suggests that the portrait of a successful BCI user can depend on handedness.

There is evidence that personality traits differ in right- and left-handers. Left-handers are significantly more agreeable [53] and more emotionally unstable [54], more dominant and less nurturance [55]. As to extraversion, early study [56] demonstrated that right-handers are more extraverted compared to left-handers (only women, not men), however, the results of modern research studies are directly opposite [53]. Another modern study [57] does not reveal any difference between left- and right-handers but demonstrates that the left-handers are more extraverted than the right-handers according to self-estimation of the level of extraversion. The study [57] demonstrates that both left- and right-handers are more extraverted than ambidextrous people. These differences among the studies mentioned above are apparently connected with the size and other characteristics of samples, and with the tests used for handedness estimation. Modern studies suggest that it is more meaningful to divide humans into strong- versus mixed- handed (consistent- versus inconsistent (ambidextrous) handed), than to left– and right-handed [58].

Some people are so-called latent left-handers. The term “latent left-handedness” was used by A.R. Luria for people who were left-handed in the Napoleon’s pose (upper left forearm) and finger grip (upper left thumb) tests but used their right hand to write and hold cutlery [59]. So, latent left-handers demonstrate inconsistent (mixed) handedness. A modern study carried out on a large group of volunteers (7562 people) showed that the position of the right forearm and right finger on top prevails in right-handers, vice versa in left-handers and in mixed type [60].

It is not known how “handedness” (left-handedness, latent left-handedness, consistent/inconsistent handedness) affects the success of BCI control, and how it relates to personality traits. At the same time, this factor can predict the success of BCI control and influence the success of neurorehabilitation with BCI, based on the classification of brain states during kinesthetic imagination of movements.

The aim of this study was to investigate the correlations between personality traits and accuracy of brain states classification during imagination of the right or the left hand in naive right-handers with different degrees of handedness (“pure” right-handers and latent left-handers).

## 2. Materials and Methods

### 2.1. Participants

Forty-four healthy BCI-naive subjects took part in the study: 19 men, 25 women, age range 19–25 years. All volunteers were right-handed [61], 18 from 44 were characterized by latent left-handedness [59]. Participants were required to have full contractual capability and no neurological disease.

The study was conducted according to the protocol of motor imagery BCI clinical evaluation approved by the Ethical Committee of the Research Center of Neurology (#12/14 of 10 December 2014) as a part of clinical trials registered at clinicaltrials.gov (accessed on 18 June 2021) (“iMove,” trial number NCT02325947).

### 2.2. Measures

All subjects were tested by Sixteen Personality Factor Questionnaire by Cattell (187 questions) and evaluated the rate of subjective complexity of the right and the left hand movement on a Likert scale from 1 to 5, where 1—very easy, 2—easy, 3—neutral, 4—difficult, 5—very difficult.

Latent left-handedness was detected using the Bragina-Dobrohotova method [62] based on [59]. It included three tasks:

Clapping: participants were requested to clap. The preference was determined by the upper hand.

Hand clasping: participants were requested to clasp their hands with the fingers interlaced. The preference was determined by the thumb «on-top» position.

Participants were requested to close their eyes and stretch arms out in front of them. The preference was determined by the arm that is higher.

The hand asymmetry coefficient (AC) (Equation (1)) was calculated as follows:

(1) $$\mathrm{AC}=\frac{R-L}{R+L+N}$$

where R is the number of times the right hand was a preference, L—the left, N—no hand was a preference. Subjects with a negative AC value were considered latent left-handers, with positive AC—as “pure” right-handers.

EEG was recorded using a 24 channels SmartBCI wireless electroencephalograph (produced by Aliot, St. Petersburg, Russia). The electroencephalograph (weight 50 g) was attached close to point of contact of electrodes with the surface of the head.

### 2.3. Experimental Procedure

During the experiment session the participants were sitting in a comfortable chair with arms lying relaxed on knees, approximately 1 m away from a computer screen.

#### 2.3.1. A Session of Control of BCI Based on Kinesthetic Imagination of Hand Movements

The participants were instructed to imagine kinesthetic (not visual) sensations during lifting and lowering the RH or LH from knee to level of their shoulder. Symbols appeared in random order on the monitor screen for 10 s, according to which subjects had to imagine movements of RH or LH or to be at the rest state.

The subjects were asked to perform motor imagery with LH or RH or be at the rest according to the instructions appearing on the screen. An icon then highlighted on the left or right side of the screen for 10 s to instruct the subjects to use their LH or RH to perform motor imagery. When none of the icons were highlighted, the subjects were instructed to be at rest. The order of highlighting of the left or right icons or its absence was randomized. Motor imagery included imagining kinesthetic (not visual) sensations when raising and lowering RH or LH from knee to level of their shoulder.

#### 2.3.2. Motor Test

The motor test included real and imagery lifting and lowering RH or LH from knee to level of shoulder for 3 min for each hand. Real hand movements were carried out according to the vertical motion of the arrow on screen, imagery movement at the same pace without visual stimuli.

After each stage of the motor test, participants evaluated the rate of subjective complexity of the stage on a Likert scale from 1 to 5, where 1—very easy, 2—easy, 3—neutral, 4—difficult, 5—very difficult.

### 2.4. EEG Analysis

EEG signals were analyzed to classify states for the pairwise asynchronous BCI based on desynchronization of the sensorimotor rhythm and to estimate accuracy of classification of brain signals when imagining movements occurred. Three brain states: imagery of LH movement (1), imagery of RH movement (2), at the rest (3) were analyzed in pairs: 1 vs. 2, 1 vs. 3, and 2 vs. 3. Classification accuracy was assessed by cross-validation, i.e., iterative partitioning of each pair of states into the training and testing sets, with the classifier trained using the training set (the duration of training the classifier was about 3 min) and the testing set being used to evaluate classification quality (offline). The accuracy of the classification was evaluated by the probability of recognizing exactly the mental state that was specified by the instruction. In case of random recognition of two states, this probability is equal to 0.5. Thus, the classification accuracy of brain states was assessed during imagination of the right hand (RH) in comparison with the rest state (ARH), the left hand (LH) in comparison with the rest state (ALH) and of RH in comparison with LH (ARLH).

### 2.5. Statistical Analysis

The dependence accuracy of brain states recognition on personality traits (as well as on the subjective complexity) was performed by methods of correlation and factor analysis. The nonparametric Mann–Whitney test was used to assess the reliability of differences between subgroups of subjects with high and low values of each of personality traits. Since the samples were not very big, and not all distributions were normal, the Spearman rank correlation coefficient (nonparametric) was calculated during the correlation analysis, and in addition, the data were analyzed using the parametric Pearson test.

## 3. Results

### 3.1. Differences between Accuracy of Classification of Brain Activity during Imagination of RH or LH Movements Depending on Personality Traits without Taking into Account Latent Left-Handedness

The average classification accuracy of brain states was 65.6% ± 8.3% (m ± SD). The classification accuracy of brain states during imagination of the right or the left hand in comparison with the rest state (ARH, ALH) was significantly (*p* < 0.05) higher, then one of the right hand in comparison with the left hand (ARLH) (66.2% ± 9.1%, 67.7% ± 8%, and 62.9% ± 7%, respectively). The minimum classification accuracy was 49.5%, which does not exceed the chance-level performance (1 case from total 132), the maximum one was 90%.

**Figure 1:**
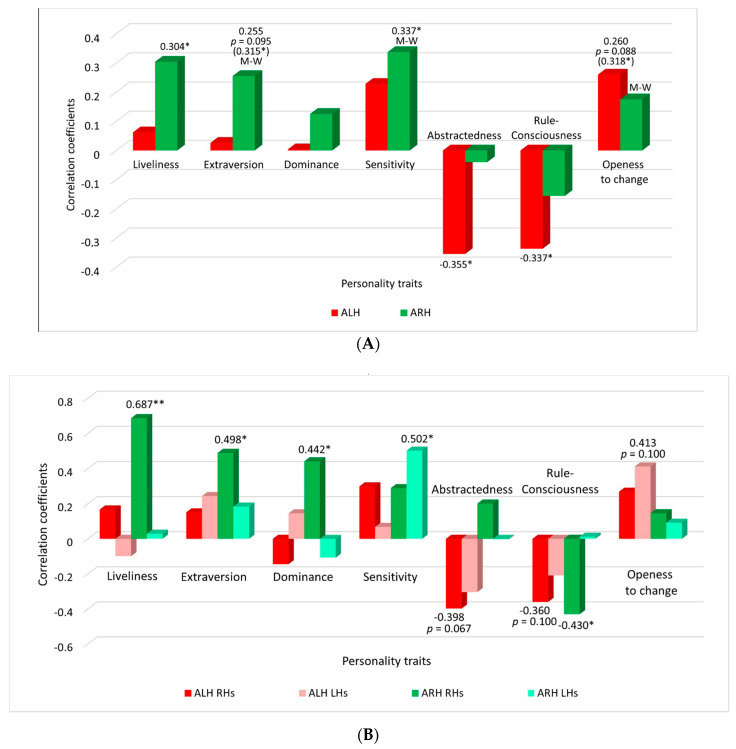
Nonparametric (Spearman’s) correlation coefficients between personality traits and the accuracy of classification of brain signals when imagining movements of the right (ARH, green columns) and left hand (ALH, red columns) in comparison to the rest. (**A**)—data of all participants (“pure” right-handers and latent left-handers together), (**B**)—data of “pure” right-handers (RHs) and latent left-handers (LHs) separately. Significant correlations are marked with an asterisk (*) (*p* < 0.05) or two asterisks (**) (for *p* < 0.01), *p*-value is indicated in a case of trend. Significant (*p* < 0.05) differences between subgroups with a high and low value of the factor according to Mann–Whitney test are marked with M-W.

Significant correlations between the personality traits and ARH or ALH are shown in Table 1, Appendix A, and Figure 1A.

ARH significantly positively correlates with F2 (extraversion), F (liveliness) and I (sensitivity). The mean values ARH for subgroups with higher and lower values of factors F2 and I, significantly differ (according to Mann–Whitney test)—classification accuracy is higher in subgroups with higher values of these features. Consequently, imagination of movements of RH is more successful in extroverts than in introverts; in people who are more reckless, spontaneous, and expressive than those who are reserved, serious, self-absorbed; in more sensitive and intuitive people than in tough, self-confident, serious utilitarian.

Besides for Factor I (sensitivity) it was shown significant negative correlation (r = −0.332) with ARLH. This means that in more sensitive individuals brain states in imagining LH and RH movements differ less than in less sensitive individuals, although, as mentioned above, sensitivity is positively associated with ARH.

ALH does not depend significantly on the factors mentioned above, but it is significantly negatively correlated with factors M (abstractedness) and G (rule-consciousness). This means that imagination of LH movements is more successful in practical realists than in people who are prone to abstract thinking; in rule-ignoring non-conformists and skeptics than in conscientious obedient moralists and conservatives. There is also a tendency (*p* = 0.069) of significance of differences between subgroups with higher and lower values of factor G according to Mann–Whitney test.

As well as factor G (rule-consciousness) factor Q1 (openness to change) revealed a significant correlation with ALH, as well as significant difference between ARH in the subgroups with higher and lower values of this trait according to Mann–Whitney test (*p* < 0.05). This means that the individuals open to change, inclined to experiment, analysis, criticism, liberalism, free thinking, and flexibility better imagine both RH and LH movements than traditionalists and conservatives.

To clarify “portraits” of users who are able to more successfully control BCI based on imagery movements of RH and LH, a factor analysis of personality traits of subjects was carried out. The result of the analysis 7 factors was revealed that explain 71% of the variance.

New variables were obtained corresponding to the values of these new factors. These new variables were correlated with classification accuracy parameters. Correlation analysis revealed a significant (*p* < 0.01) negative relationship of one of the factors with ALH. This factor includes the following traits estimated by Cattell Questionnaire (factor loading and its sign are indicated in parentheses): abstractedness (M; −0.752) (grounded, practical, prosaic, solution oriented, steady, conventional), privateness (N; +0.670) (private, discreet, non-disclosing, shrewd, polished, worldly, astute, diplomatic) and warmth (A; −0.403) (impersonal, distant, cool, reserved, detached, formal, aloof). It means that ALH (but not ARH) is higher in practical, private, low social (the latter with a lower factor load) individuals.

It should be noted that, since samples were not very large, data obtained using factor analysis are less reliable than paired correlations, but they do not contradict results of correlation analysis and allow us to clarify the “portrait” of a successful BCI user performing a task for the first time.

Thus, the results of testing of all the participants (“pure” right-handed and latent left-handed individuals together) shows that practical, reserved, skeptical and not very sociable people are more successful in imagining LH movements (brain state differ higher from the rest), expressive sensitive extroverts are more successful in imagining RH movements, opened to change individuals—in imagination of movement of both RH and of LH.

### 3.2. Differences between Accuracy of Classification of Brain Activity during Imagination of RH or LH Movements Depending on Personality Traits Taking into Account Latent Left-Handedness

The average classification accuracy of states for “pure” right-handers is 66 ± 8.2%, for latent left-handers—65 ± 8.5% (Table 2). There are no significant differences in ARH, ALH and ARLH between the “pure” right-handers and latent left-handers groups. The minimum classification accuracy in “pure” right-handers group is 49.5%, which does not exceed the chance-level performance (1 case from 54 total), the maximum one is 90%; in the group of latent left-handers—52.1% (which exceeds the chance-level performance) and 89.1%, respectively. ARH is significantly higher than ARLH within the group of “pure” right-handers only.

Data (Table 3, Figure 1B) shows that liveliness (F) and extraversion (F2) significantly positively correlate with ARH in “pure” right-handers, but not in latent left-handers. The correlation coefficients are about twice as high in “pure” right-handers compared to the group of all the subjects. Two factors—dominance (E) and rule-consciousness (G), also demonstrate significant correlation with ARH in “pure” right-handers, but not in latent left-handers (Table 3). Although values of rule-consciousness (G) does not correlate significantly with ARH in the group of all subjects (see Table 1), there is a tendency of differences between ARH in subgroups with higher and lower values of this factor. Now it can be realized that in “pure” right-handers there are significant correlations both ARH, and ALH with values of rule-consciousness (G).

Correlations of factors abstractedness (M) and openness to change (Q1) do not depend on latent left-handedness. The factor I (sensitivity) significantly positive correlates with ARH in latent left-handers, but not in “pure” right-handers. The correlation coefficient is higher in latent left-handers (+0.502) compared to the group of all the subjects (+0.337) (Table 3).

Furthermore, Factor I (sensitivity) demonstrates negative significant correlation with the ARLH (r = −0.615) in latent left-handers (the correlation is also significant, but less in the group of all the subjects (r = −0.332), and it is not significant in “pure” right-handers). Other personality traits do not correlate significantly with ARLH.

Factor analysis of personality traits was carried out in groups of “pure” right-handers and latent left-handers. New variables were obtained that correspond to the values of new factors identified as a result of the factor analysis. These new variables were correlated with the values of classification accuracy. This correlation analysis revealed the following significant (*p* < 0.01) correlations (correlation coefficients, factor loadings and their sign are indicated in parentheses).

In “pure” right-handers:

positive correlation (r = +0.525 *) of ARH with factor including following traits estimated by Cattell Questionnaire: extraversion (F2; +0.822), openness to change (Q1; +0.804), dominance (E; +0.7), liveliness (F; +0.696);

negative correlation (r = −0.553 **) ALH and positive correlation (r = +0.442 *) of ARLH with factor including two traits estimated by Cattell Questionnaire: abstractedness (M; +0.942) and privateness (N; −0.649).

In latent left-handers:

negative correlation (r = −0.498 *) of ARLH with factor including the following traits estimated by Cattell Questionnaire: rule-consciousness (G; +0.724) and sensitivity (I; +0.696).

Thus, the accuracy of classification is to a greater extent connected with personality traits in “pure” right-handers during the imagination of RH movements. The accuracy of recognition of brain states in this case is influenced by a wide range of personality traits, including extraversion (F2), openness to change (Q1), dominance (E), and liveliness (F). Abstractedness (M) and privateness (N) affect the imagination of LH movements by “pure” right-handers. In latent left-handers, sensitivity (I) and rule-consciousness (G) influence the accuracy of recognition of brain states during imagination movements of RH comparative to LH (not comparative to the rest, as in right-handers). It is necessary to emphasize that, since samples were not very large, data obtained using factor analysis are less reliable than paired correlations.

### 3.3. Subjective Complexity of Real and Imaging Movements Correlated with the Accuracy of Classification of the Brain States

The average values of subjective complexity (Table 4) show that it is subjectively more difficult to imagine movements than to perform them (on average by 37.5% for the group of all the subjects (*p* < 0.001); by 41% for “pure” right-handers (*p* < 0.005); by 32% for latent left-handers (*p* < 0.01)). A more detailed analysis shows that it is subjectively more difficult to imagine LH movements than to perform them, both in the group of all subjects (by 45%, *p* < 0.001), and in “pure” right-handers (by 54%, *p* < 0.01), and in latent left-handers (by 33.5%, *p* < 0.05), while subjective complexity of real and imagery movements of RH differs significantly only for the group of all subjects (by 30%, *p* < 0.05). There were no significant differences in subjective complexity of both real and imagery movements of RH and LH.

**Figure 2:**
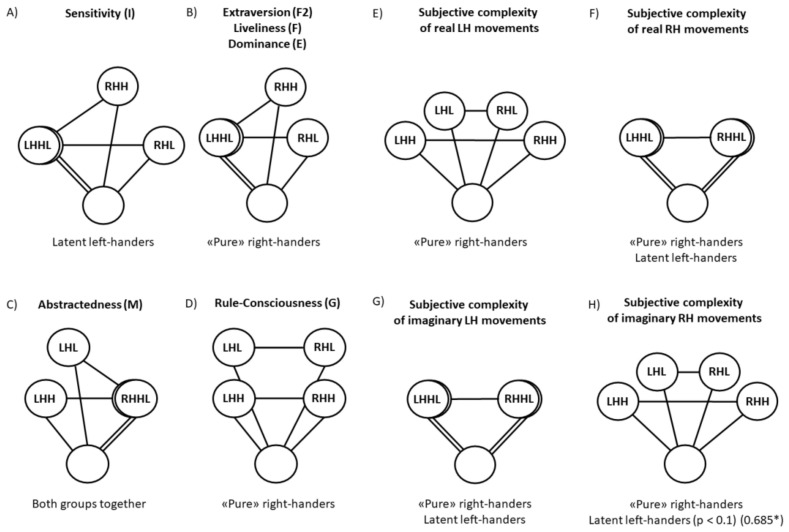
Scheme of the mutual location of areas of state of parameters calculated from EEG characteristics and used for classification, characterizing different brain states: at the rest (empty circles), while imagining movements of the right hand (RH) or left hand (LH) with low (L) or a high (H) value of an individual personality trait or subjective complexity of real or imaginary movements among “pure” right-handers, latent left-handers and both groups together. The distance between circles corresponds to the classification accuracy of brain states. *p*-value and Pearson correlation coefficients (in parenthesis with an asterisk) are indicated in a case of Spearman trend (Figure 2H). The description and explanations for the figure are given in the text.

Analysis of correlations of subjective complexity with classification accuracy does not reveal significant correlations of subjective complexity with ARH and ALH for the group as a whole and for groups of “pure” right-handers and latent left-handers. However, ARLH significantly correlates with subjective complexity of imaginary movements of RH (but not the LH), in group as a whole (r = 0.697, Table 4), as well as in group of “pure” right-handed (r = 0.664, Table 4, Figure 2); for group of latent left-handers tendency in the same direction. Besides that, ARLH significantly correlates with subjective complexity of real LH (but not RH) movements in groups of “pure” right- handers.

## 4. Discussion

The results of the study revealed a correlation between the accuracy of classification of brain signals during imagination of RH or LH movements with personality traits and subjective complexity of real and imaginary movements, and the relationship between these factors can change depending on the latent left-handedness. Latent left-handedness is a variant of mixed (inconsistent) handedness, which, according to modern researchers, is more significant for dividing people according to the degree of left-handedness than for left-handers and right-handers [58]. All the data obtained are assumed to be associated with laterality—differences in information processing and motor control in the right and left hemispheres of right-handers and left-handers.

In right-handers the left dominant hemisphere comparative to their right one is characterized by shorter connections between neurons [63], more local information processing [64], higher blood circulation [65], more developed sensory and motor areas [66]. It is specialized in verbal cognition, gestures, fine motor skills, and control of sequence of movements, while the right-hemisphere in right-handers—in nonverbal cognition, postural control and positional aspects of movements [67,68,69,70]. When controlling hand movements, dominant hemisphere is specialized in limb trajectory control, and nondominant—in limb position control [71,72,73,74,75,76,77,78,79,80,81,82,83,84,85,86,87].

The left-handers are characterized by a smaller morphological and functional interhemispheric asymmetry comparative to the right-handers [67,69,70], which is apparently connected with their more pronounced interhemispheric connections through corpus callosum [88,89,90]. There is evidence of a greater strength of connections between the occipital regions of the right and left hemispheres (coherence) of left-handers than right-handers [91]. Left-handers are characterized by less asymmetry of the central sulcus [92] and of the cerebellum [93] than right-handers. A significant correlation was found between the degree of handedness and the size of the planum temporale, assessed by the bifurcation of the Sylvian fissure [94]. During RH and LH movements EEG analysis revealed differences in right- and left-handers not only in interhemispheric interactions, but also in cortico-subcortical ones [95].

Motor control in right-and left-handers is characterized by two features. On the one hand, when organizing the movements of right-handers and left-handers, there is often “mirror” activity in the dominant and subdominant hemispheres. In both right-handers and left-handers, the contralateral areas of the cortex are activated more than the ipsilateral areas [96], and this is more pronounced for the dominant hand than for the non-dominant hand (non-dominant hand demonstrates more bilateral pattern of activity [97,98]. The non-dominant hand is better in the task of comparing proprioceptive goals than the dominant hand: RH in left-handers and LH in right-handers [99]. The study of the accuracy of hand movements when reproducing sequences modified according to the principle of position constancy suggests that the subdominant hemisphere of both right-handers and left-handers remembers and uses information about positions, despite the change in the movements themselves [86].When reproducing sequences modified by the principle of constancy of the amplitude and direction of movement (and changing the position), the dominant hemisphere in both right-handers and left-handers remembers and uses information about the parameters of the movements [87].

On the other hand, even during a simple movement such as the clenching of the fist, with a basic “mirror” similarity of the activated brain areas in right- and left-handers, there are differences in the strength of connections between different areas [100,101]. During finger movement of the dominant hand the contralateral primary motor cortex of the dominant hemisphere is activated in both right- and left-handers, but there is no “mirroring” for other motor areas [100]. Based on the data that both in right- and left-handers RH reproduced simple rhythmic patterns more precisely than LH, it was suggested that the left hemisphere control the serial organization of movements regardless of the handedness [102]. During LH movements left dorsal premotor cortex is more active both in right- and left-handers, and this asymmetry is less in left-handed people [103]. Reproducing of grasping force is asymmetrical in right-handers, but not in left-handers [104,105].

In general, for right- and left-handers “mirror” brain patterns are more typical for more simple movements, whereas during more complex or sequential movements the brain patterns are more complicated, including left premotor cortex, however this asymmetry is not so pronounced in left-handers [70,79,100,106,107].

Taking into account these data, we will answer the questions that arise when considering the results of the study.

### 4.1. Why Are Lively Dominant Extroverts Higher in ARH?

It is known that the left nigrostriatal dopaminergic system prevails in people with a dominant RH, and vice versa (right nigrostriatal dopaminergic system) in left-handers [108]. At the same time, there is information about the predominant content of dopamine in basal ganglia (in globus pallidus, caudate nucleus, and putamen) of the left hemisphere compared to the right hemisphere [109,110]. Extroverts have larger dopaminergic activity than introverts [111]. Dopamine plays the role of a stimulating neurotransmitter in the nigrostriatal dopaminergic system that helps to increase motor activity, reduce motor inhibition, and muscle hypertonicity. This role of dopamine apparently leads to disinhibition of neuronal pools associated with regulation of movements and motor imagery. Globus pallidum regulates complex motor acts; when it is irritated, contraction of limb muscles is observed. Caudate nucleus is important in conscious control of motor activity, and putamen is responsible for regulation of movement and influences various types of learning. The level of basal ganglia is the level of synergies and stereotypes according to N.A. Bernstein [112,113,114], and movements that our subjects imagined during the experiment (lifting and lowering arm) are well-learned, stereotyped movements that can be categorized as “stereotypes”. Therefore, it can be assumed that ARH, but not ALH, was higher in extraverts (compared to introverts) due to the greater activity of their dopaminergic system.

As to other traits that revealed a correlation with ARH in “pure” right-handers—liveliness (F) and dominance (E)—there is no direct information in the literature, however, these personality traits consistently complement the image of the user-extrovert who more successfully controls BCI when imagining RH movements.

### 4.2. Why Does the Factor Sensitivity (I) in Latent Left-Handers (But Not in “Pure” Right-Handers) Affect ARH (Not ALH)?

It is known that when organizing movements, left-handers are characterized by “mirroring”, that is, when performing movements, the dominant hemisphere of both left-handers and right-handers regulates work of the dominant hand and controls ballistic movements, and the subdominant hemisphere provides a description of positions (positional coding) using feedbacks when organizing movements: the non-dominant hand copes better with positional tasks—RH in left-handers and LH—in right-handers [71,72,73,79,80,81,82,86,87,99,115]. It seems likely that the higher sensitivity (I), the greater the inflow of afferent kinesthetic information that provides feedbacks, and feedbacks, as mentioned above, are used to a greater extent when organizing movements by the right hemisphere of right-handers and the left hemisphere of left-handed ones. Therefore, in our experiment, activity of the brain during imagination of movements of RH is more different from the rest in latent left-handers with greater sensitivity (I).

It would be logical to expect that also in “pure” right-handers with a higher sensitivity (I), the classification accuracy in imagining LH movements would be higher, but in this case no significant changes were found. Perhaps there are some additional factors. It is supposed to be connected with the differences in the morphofunctional organization of the brain of right-handers and left-handers. These differences reveal themselves in data as follows. Even during such an elementary movement as making a fist a fundamental “mirror” similarity of the activated areas of the brain of right-handers and left-handers is observed, but there are differences in strength of connections between different areas [100]. Reproduction of force grip is asymmetric in right-handers, but symmetric in left-handers [104,105]. EEG analysis during the movements of RH and LH reveals differences in right-handers and left-handers not only in interhemispheric, but also in cortical-subcortical interactions [116]. Studying RH and LH movement sequences in right- and left-handers demonstrates that during the first reproduction of a memorized sequence of movements, errors are “mirrored”. However, when the same sequence is reproduced with the opposite hand (skill transfer), “mirroring” is lost and the errors depend on other aspects, which are nevertheless also associated with laterality [87].

### 4.3. Why Does Factor Abstractedness (M) Affect ALH?

In the group of “pure” right-handers and latent left-handers together abstractedness (M) is negatively correlated with ALH, i.e., in more “concrete” subjects, brain signals during LH movement imagination differ more from the rest state. It can be assumed that this is due to fact that the right hemisphere of right-handers specializes in holistic descriptions, in formation of an internal representation of the “body scheme”; it provides regulation of posture and movement, carried out with feedback, and positional aspects of movement [71,72,73,79,80,81,82,115]. On the other hand, the left hemisphere is characterized by more local information processing (shorter interhemispheric connections [63], it provides regulation of ballistic movements (without feedback) and their dynamic aspects [71,72,73,79,80,81,82,115], fine motor skills, gestures, sequences of movements, and speech, categorization [117], which requires abstraction. This information about interhemispheric asymmetry can be compared with such personal factors as concreteness and abstractness. Concreteness—taking into account holistic sensory information, including kinesthetic information obtained from feedbacks during movements, abstractness—ignoring details, truncated information, ballistic movements, descriptions in a language when a word reflects a set of characteristic features, categorization (e.g., “tree”—roots, trunk and branches, “hare”—moves, jumps, ears). From this point of view, more concrete, practical and less abstract people have more activity in the areas of the brain associated with organization of LH movements, which is more different from activity at the rest, which affects (manifested in) an increase in the classification accuracy.

Why is latent left-handedness not a factor that determines the correlation between abstractedness (M) and ALH? It can be assumed that this is due to the fact that the right hemisphere in right-handers and left-handers works more similarly than the left. It can be assumed that this is due to the fact that the right hemisphere in right-handers and left-handers works more similarly than the left. Right-handers differ more in left and right hemispheres than left-handers [67,90,92,93]. Depending on the degree of left-handedness, properties of the left hemisphere change to a greater extent, not the right hemisphere [70]. All these features are associated with the nature of organization of intra- and interhemispheric connections. In right-handers, intrahemispheric connections in the left hemisphere are shorter [63] than in the right; in left-handers, interhemispheric connections are more pronounced, due to which lateralization of functions is not so pronounced. When imagining LH movements, the right hemisphere is more active, which differs less between right-handers and left-handers, and therefore we do not observe dependence of the classification accuracy on latent left-handedness.

### 4.4. What Is the Reason for the Differences in the Subjective Complexity of Realizing Real Movements of the RH and LH and Their Imagination?

Subjective complexity is a parameter that is evaluated by the subject based on their own feelings, not too often used in research. This approach was used to determine the ability to imagine movements in patients with movement disorders, where it was shown that the brightness of movement imagination correlates with the activation of motor cortical zones, and in patients it is less than in healthy ones [118]. The complexity of the new motor imaging paradigm compared to the traditional one was evaluated by [119] when studying the effect of long-term seven-day training on the subjective difficulty of imagining movements and the level of pain in patients with spinal cord injuries [120]. This method was also used to compare the subjective complexity with the task’s index of difficulty and with the movement time [121,122,123]. We failed to find studies on correlation between the accuracy of brain states classification and the subjective complexity of the performance of real hand movements or their imagination.

Our experiments have demonstrated that in “pure” right-handers the subjective complexity of real LH (not RH) movements and the subjective complexity of imagery RH (not LH) movements correlate with ARLH. In addition, subjective complexity of real and imaginary RH and LH movements is associated with different personality traits. It can be assumed that those individuals for whom it is subjectively difficult to make a real movement pay more attention to this process. Since kinesthetic feedbacks play a greater role in motor control in the right hemisphere than in the left one [71,72,73,78,79,80,81,82,115], greater attention to kinesthetic information plays a greater role in the LH real movements.

Why is ARLH greater in those who find it subjectively more difficult to imagine the movements of the RH, and not the LH? We can assume that this is also due to attention to the task—the more difficult the task, the more attention, and attention disinhibits the specific loci of the brain involved. But why is this observed only for RH, but not for LH? Here, apparently, the answer lies in the differences in the organization of movements—ballistic in the left hemisphere and positional—in the right one [71,72,73,74,75,76,77,78,79,80,81,82,84,86,87,115]. When imagining movements, feedbacks are not involved, therefore, the factor of complexity of imagery movements connected with the left hemisphere and with the imagination of RH movements in “pure” right-handers (in latent left-handers only a tendency, *p* < 0.1).

Why do personality traits affect the accuracy of the classification of brain states in imagining the hand compared to rest, and subjective complexity affects the accuracy of the classification of brain states in imagining the right and left hands (but not compared to rest)? Probably, there is some parameter that determines the differences between LH-RH, but it does not affect the differences between LH-rest and RH-rest. It can be assumed that this parameter is determined by the severity of interhemispheric connections. A significant role in motor behavior is played by symmetrical movements, which are carried out simultaneously by LH and RH. The realization of these movements is provided by the simultaneous activity of the loci that control the movements of RH and LH. In the case of high subjective complexity of the movement of a particular hand, and, as it seems likely, more attention to the task, the activity of the locus controlling the movements of this hand will be greater. Then the differences between the activity of areas which control the symmetric RH and LH will be more, and more the classification accuracy ARLH, but not ARH and ALH.

In latent left-handers, apparently, the differences between brain activity in the organization of symmetrical hand movements are less (the connections between areas are less asymmetric in left-handers, i.e., their hand control is more bilateral [101]). This suggests that the differences between the activity of the brain in the organization of symmetrical hand movements are less in latent left-handers than in pure right-handers. That is why the correlation between the accuracy of classification ARLH and the subjective complexity is not significant in latent left-handers.

### 4.5. How Can We Imagine the Relationship between the Results Described above?

The study showed that different personality traits and subjective complexity are differently correlate with the accuracy of the classification, evaluated in different ways (ARH, ALH, or ARLH). At first glance, some of the results obtained seem paradoxical. How, for example, is it possible that sensitivity in latent left-handers correlates positively with ARH and negatively with ARLH? In order to imagine this, we propose a hypothetical scheme that demonstrates how it can be represented in the space of EEG parameters used to classify brain signals in different states (Figure 2A).

Figure 2 shows the relationship between the EEG parameters that characterize the brain state under different conditions—when imagining the movements of RH, LH and at the rest. On Figure 2A the area of states of EEG parameters corresponding to the state of the brain when imagining RH movements in the subgroup of individuals with a high (H) levels of the factor sensitivity (I) (RHH) is located at a further distance from area at the rest state (empty circle) than in subgroup of individuals with a low (L) expression of these personality trait (RHL). This reflects the data on significant correlation between ARH and this factor. The absence of correlations between ALH is reflected in the scheme by the coincidence of the positions of the circles reflecting these parameters in individuals with high and low (LHHL) values of this factor. The smaller LHHL-RHH than LHHL-RHL distance reflects the differences in EEG parameters when imagining the movements of RH and LH in subgroups with high and low level of the factor under consideration, which is manifested in the presence of a negative correlation between this factor and ARLH.

This scheme can also be applied to other data obtained. Figure 2B illustrates the area of states of EEG parameters in “pure” right-handers with high and low extroversion, expressiveness, and dominance. The area of states of EEG parameters corresponding to the state of the brain when imagining RH movements in individuals with a high level of these personality characteristics (RHH) is located at a more distant distance from the area corresponding to the state of the rest (empty circle) than in individuals with a low expression of these personality traits (RHL). This reflects a significant correlation of the accuracy of classification ARH with these personality traits. The absence of significant correlations between the accuracy of classification ALH and these traits is reflected in the scheme by the coincidence of the positions of the circles reflecting these parameters in individuals with high and low (LHHL) levels of these personality traits. The same LHHL-RHH and LHHL-RHL distances reflect the absence of correlations between the discussed personality traits and the accuracy of classification ARLH.

The accuracy of classification of brain signals when imagining LH movements in comparison with rest (ALH) is affected by the factor abstractedness (M). Abstractness is negatively correlated with ALH, i.e., in more “practical” subjects the brain signals when imagining LH movements are more different from signals in the rest state. This is reflected in Figure 2C, where the area of states of the EEG parameters corresponding to the states of the brain when imagining LH in individuals with a low level of abstractedness (LHL) is at a further distance from area corresponding to the rest state (an empty circle) than in individuals with a high expression of this factor (LHH). This reflects a significant correlation between ALH and this factor. The absence of significant correlations between ARH and this factor is reflected in the scheme by the coincidence of the positions of the circles reflecting these parameters in individuals with high and low (RHHL) levels of this factor (there is no significant correlation of ARH and abstractedness). The same RHHL-LHH and RHHL-LHL distances reflect the absence of significant correlations between this factor and the accuracy of classification ARLH.

The influence of the factor rule-consciousness (G) on the accuracy of state recognition when imagining movements of both RH and LH compared to the rest state (both ARH and ALH) is illustrated by Figure 2D. For pure right-handers, this factor G significantly correlates with ARH and ALH, for the group of all subjects, the factor G, as the factor Q1 (openness to change), significantly correlates with ALH, and there are also significant differences in ARH in subgroups with high and low values of this trait according to the Mann–Whitney criterion (*p* < 0.05).

As can be seen from the schemes in Figure 2A–D, the relative position of the areas of brain state in the space of EEG parameters differs depending on personality traits, on the imaginary hand (RH or LH), and on the factor of latent left-handedness.

In the right part of Figure 2 (Figure 2E–H), schemes represent the areas of the brain states in the space of EEG-parameters, which change depending on the subjective complexity of real movements and their imagination. In “pure” right-handers and latent left-handers subjective complexity of real movements of RH and of imagery movements of LH is not correlated with the classification accuracy of brain states. It is shown by coincidence of the circles RHH and RHL (signed by abbreviation RHHL) for real movements and by coincidence of the circles LHH and LHL (signed by abbreviation LHHL) for imagery movements, as well as by the same distance between empty circle (the rest state) and matching circles (RHHL for real movements or LHHL for imagery movements). The subjective complexity of LH real movements and of RH imagery movements in ”pure” right-handers correlates with ARLH, which is reflected in the greater distance LHH-RHH, than LHL-RHL. At the same time, the distances between the empty circle (the rest state) and the circles of all other states are the same, which reflects the absence of correlations of subjective complexity with ARH and ALH.

## 5. Conclusions

The study found that the relationship between brain signal recognition and personality traits depends on whether an individual imagines RH or LH movements, as well as whether they are “pure” right-handed or latent left-handers. Such personality characteristics as extraversion, liveliness, dominance, and sensitivity are significant for the imagination of RH movements, and the first three are significant for the group of “pure” right-handers, and the last one is significant only for hidden left-handers. For the imagination of the movements of the LH, practicability (negative correlation ALH with factor abstractedness) is mainly important, and there is no dependence on the degree of handedness. Both rule-consciousness and openness to change are important for imagination of both hands. Rule-consciousness significantly negatively influences the accuracy of recognition in “pure” right-handers, not in latent left-handers. Openness-to-change positively influences the accuracy of recognition and does not depend on level of handedness.

The analysis of the subjective complexity of real and imagery movements showed that, in contrast to personality traits, subjective complexity correlates only with the accuracy of recognizing states when imagining RH and LH movements, but not states when imagining movements compared to rest. For individuals with one personal profile, it is subjectively more difficult to perform real movement, for individuals with another profile —to imagine these movements. The influence of personality traits on the subjective complexity of imagery movements was revealed only in pure right-handers.

Data obtained can be considered as the concept of laterality—differences in information processing and motor control in inter- and intra-hemispheric connections between neurons and in neurotransmitters in the right and the left hemisphere in right- and the left-handers. The differences in personality traits which are optimal for imagination of RH or LH and on the influence of latent left-handedness may be important for rehabilitation of patients after stroke.

In future studies, we are going to increase the sample size; to analyze the correlation of personality traits not only with motor imagery of the upper limbs, but of the lower limbs as well; to study the dynamics of training of the imagination of the upper and lower extremities and its correlations with personality traits and brain asymmetry.

## Figures and Tables

**Table 1:** Correlations between personality traits and the accuracy of classification of brain states during imagination of hand movements comparative to the rest (data of all 44 participants).

Personality Trait (Cattell)	Accuracy of Classification of Brain States in Imagining Left Hand Movements and in Rest State (ALH)		Accuracy of Classification of Brain States in Imagining Right Hand Movements and in Rest State (ARH)	
	PCC	NCC	PCC	NCC
Liveliness (F)	-	-	+0.346 *	+0.304 *
Extraversion (F2)	-	-	+0.315 *	+0.255 (*p* = 0.095)
Sensitivity (I)	-	-	+0.347 *	+0.337 *
Abstractedness (M)	−0.270 (*p* = 0,076)	−0.355 *	-	-
Rule- consciousness (G)	−0.362 *	−0.337 *	-	-
Openness to change (Q1)	+0.318 *	+0.260 (*p* = 0.088)	-	-

PCC—the value of the parametric correlation coefficient (Pearson), NCC—nonparametric one (Spearman). Significant correlations (*p* < 0.05) are marked with an asterisk (*), *p*-value is indicated in parenthesis in a case of trend.

**Table 2:** The average classification accuracy for latent, “pure” right-handers and all participants together (m ± SD).

	“Pure” Right-Handers	Latent Left-Handers	All Participants
Accuracy of classification of the LH movements imagination comparative to rest (ALH)	66.9 ± 8.5%	66.5 ± 10.4%	66.2 ± 9.1%
Accuracy of classification of the RH movements imagination comparative to rest (ARH)	68.7 ± 8%	65.6 ± 8.1%	67.7 ± 8%
Accuracy of classification of the imagination of RH movements comparative to LH (ARLH)	62.4 ± 7.1%	63 ± 6.9%	62.9 ± 7%
Mean	66 ± 8.2%	65 ± 8.5%	65.6 ± 8.3%

**Table 3:** Correlations between personality traits and the accuracy of classification of brain states during imagination of hand movements (of the left hand (LH) or of the right hand (RH)) comparative to the rest taking into account latent handedness.

Personality Trait (Cattell)	Accuracy of Classification of Brain States in Imagining Left Hand Movements and in Rest State (ALH)		Accuracy of Classification of Brain States in Imagining Right Hand Movements and in Rest State (ARH)	
	PCC	NCC	PCC	NCC
Liveliness (F)	-	-	All +0.346 *	All +0.304 *
	-	-	RHs +0.656 *	RHs +0.687 **
	-	-	-	-
Extraversion (F2)	-	-	All +0.315 *	All +0.255 (*p* = 0.095)
	-	-	RHs +0.420 *	RHs +0.489 *
	-	-	-	-
Dominance (E)	-	-	-	-
	-	-	-	-
	-	-	-	RHs +0.442 *
Sensitivity (I)	-	-	All +0.347 *	All +0.337 *
	-	-	-	-
	-	-	LHs +0.518 *	LHs +0.502 *
Abstractedness (M)	All −0.270 (*p* = 0.076)	All −0.355 *	-	-
	-	-	-	-
	-	-	-	-
Rule- consciousness (G)	All −0.362 * RHs −0.478 * -	All −0.337 * RHs −0.360 (*p* = 0.1) -	- RHs −0.433 * -	- RHs −0.430 * -
Openness to change (Q1)	All +0.318 * - -	All +0.260 (*p* = 0.088) - LHs 0.413 (*p* = 0.1)	- - -	- - -

All—the correlation coefficient for the group of all of the subjects, RHs—for right-handers, LHs—for left-handers. PCC—the value of the parametric correlation coefficient (Pearson), NCC—nonparametric correlation coefficient (Spearman). Significant correlations are marked with an asterisk (*) (*p* < 0.05) or two asterisks (**) (for *p* < 0.01), *p*-value is indicated in parenthesis in a case of trend.

**Table 4:** Mean values of subjective complexity (mean SC) and Spearman’s correlation coefficients (correlations) between SC of real movement or motor imagery of the right hand (RH) or left hand (LH) and the accuracy of classification of brain states during imagination of movements of the left hand compared with right hand (ARLH) in the group of all participants (all), in the group of “pure” right-handers (RHs) and latent left-handers (LHs).

	LH		RH	
	Mean SC	Correlations	Mean SC	Correlations
Real movement	All 1.88	-	All 1.96	-
	RHs 1.80	RHs 0.527 *	RHs 1.87	-
	LHs 2.00	-	LHs 2.11	-
Motor imagery	All 2.73	-	All 2.54	All 0.697 **
	RHs 2.77	-	RHs 2.40	RHs 0.664 **
	LHs 2.67	-	LHs 2.78	LHs 0.604 (*p* = 0.085) (0.685 *)

The values of correlation coefficients are indicated only in a case of significance. Significant correlations are marked with an asterisk (*) (for *p* < 0.05) or two asterisks (**) (for *p* < 0.01), *p*-value and Pearson correlation coefficients (in parenthesis) are indicated in a case of Spearman trend.

**Figure A1:**
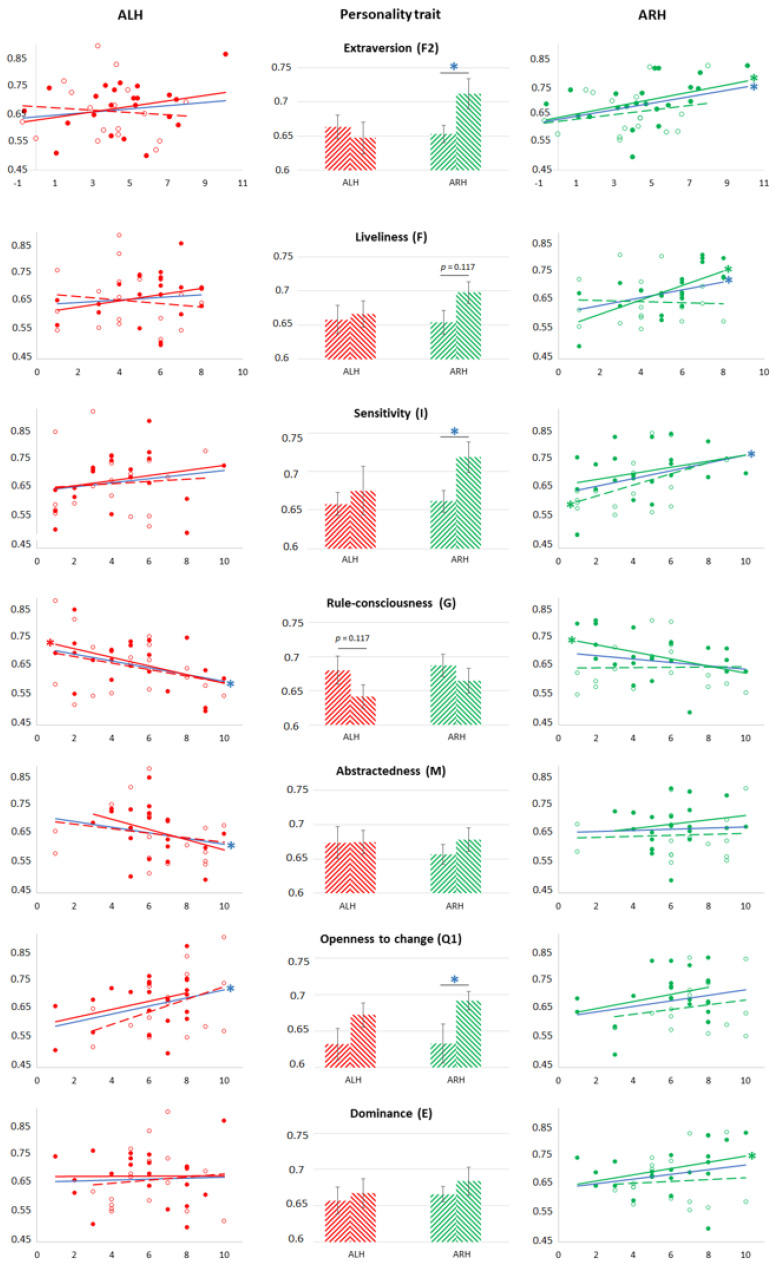
Left and right columns of plots represent the correlation fields between the factor score and the classification accuracy of brain signals during imagination of movements of LH comparative to the rest (ALH, left column) and of RH comparative to the rest (ARH, right column). Filled points and solid trend lines represent the values for “pure” right-handers; empty points and dotted trend lines represent latent left-handers. Significant Pearson’s or Spearman’s correlations (*p* < 0.05) are marked with an asterisk (*) (red asterisks stand for ALH in the group of “pure” right-handers or latent left-handers, green stand for ARH, and blue represent ALH or ARH among all participants). The factors are specified on the top of the middle plots. The middle column of the plots represents mean ALH (red columns) and mean ARH (green columns) for the subgroups of participants with the low factor score (low expression of the personality trait; left columns) and with the high score (high expression of the personality trait; right columns). Significant Mann-Whitney’s correlations (*p* < 0.05) are marked with blue asterisks (*), *p*-value is indicated in the case of trend.

## Data Availability

The datasets generated for this study are available from the corresponding author on request.
